# Heme on Pulmonary Malaria: Friend or Foe?

**DOI:** 10.3389/fimmu.2020.01835

**Published:** 2020-08-25

**Authors:** Tatiana Almeida Pádua, Mariana Conceição Souza

**Affiliations:** Laboratory of Applied Pharmacology, Institute of Drug Technology (Farmanguinhos), Oswaldo Cruz Foundation, Rio de Janeiro, Brazil

**Keywords:** hemolysis, MA-RD, *Plasmodium*, heme, severe malaria, HO-1

## Abstract

Malaria is a hemolytic disease that, in severe cases, can compromise multiple organs. Pulmonary distress is a common symptom observed in severe malaria caused by *Plasmodium vivax* or *Plasmodium falciparum*. However, biological components involved in the development of lung malaria are poorly studied. In experimental models of pulmonary malaria, it was observed that parasitized red blood cell-congested pulmonary capillaries are related to intra-alveolar hemorrhages and inflammatory cell infiltration. Thus, it is very likely that hemolysis participates in malaria-induced acute lung injury. During malaria, heme assumes different biochemical structures such as hemin and hemozoin (biocrystallized structure of heme inside *Plasmodium* sp.). Each heme-derived structure triggers a different biological effect: on the one hand, hemozoin found in lung tissue is responsible for the infiltration of inflammatory cells and consequent tissue injury; on the other hand, heme stimulates heme oxygenase-1 (HO-1) expression and CO production, which protect mice from severe malaria. In this review, we discuss the biological mechanism involved in the dual role of heme response in experimental malaria-induced acute lung injury.

## Introduction

Malaria remains one of the major public health problems. In 2018, 228 million cases and 405,000 deaths from malaria were estimated worldwide (1). Malaria is particularly prevalent in tropical and subtropical low-income regions of the world such as the African region, which accounts for 93% of the cases, followed by the Southeast Asia region with 3.4% and the Eastern Mediterranean region with 2.1% (1). The World Health Organization's (WHO) mission is to reduce global malaria mortality rates by 90% by 2030 (2). Malaria is caused by at least six known species of *Plasmodium* infecting humans: *Plasmodium falciparum, Plasmodium vivax, Plasmodium malariae, Plasmodium ovale, Plasmodium knowlesi* (3), and the more recently described *Plasmodium simium* (4). Its transmission occurs by female anopheles mosquito bites, transfusion of infected blood, or transplacentally, from infected mother to fetus [reviewed in (5)]. The vast majority of human malaria worldwide is uncomplicated resulting in fever, and factors involved in disease complications are unknown.

Severe malaria is a complication that affects multiple organs, including lungs (6). (7) reviewed the incidence of lung dysfunction in malaria patients and showed data ranging from 2 to 29%. The wide range is related to different methods to diagnose dysfunction severity. Considering the classification of pulmonary complications, it is worth mentioning that malaria is most prevalent in poor countries where methods of diagnoses, documentation, and reporting are weak. Furthermore, a large proportion of severe malaria illnesses and deaths occur in people's homes without coming to the attention of a formal health service. In accordance, although the Berlin definition is a robust and reproducible tool for identifying acute respiratory distress syndrome (ARDS), it could not be applied in low-income countries because of inaccessibility of mechanical ventilators, arterial blood gas diagnostics, and chest radiography. For instance, none of the patients with malaria in the studies of Leopold et al. (8) could be diagnosed with ARDS using the conventional Berlin definition because the requirements for positive end-expiratory pressure criteria were not known since patients were managed outside of the intensive care unit (ICU). This limitation could have the unintended consequence of underestimating and undertreating the burden of malaria-induced ARDS in many countries (9). Thus, available data concerning malaria induced-ARDS incidence worldwide may be underestimated. Herein, we use the term malaria-induced respiratory distress (MA-RD) to present studies in which ARDS has not been formally diagnosed.

Almost all *Plasmodium* species that infect humans can induce MA-RD [reviewed in (10)], including *P. malariae* (11), *P. ovale* (12), and *P. knowlesi* (13); however, this syndrome is more common in *P. falciparum* and *P. vivax* malaria (14–17). MA-RD can be observed at early time points after diagnosis or even when the parasitemia decreases or disappears [reviewed in (7)]. Besides, antimalarial treatment can also lead to lung dysfunction. For instance, during quinine therapy, it is possible to observe pulmonary exacerbated inflammatory response and reduced alveolar-capillary gas exchange (18). Primaquine treatment also leads to hemolysis and consequent ARDS in malaria patients that present G6PD deficiency (19).

The most common pathologies associated with MA-RD are pulmonary edema, dyspnea, reduction in the capacity of gas exchange, and increased levels of inflammatory mediators (7). Autopsies in patients who have died of severe malaria and ARDS symptoms showed pleural and pulmonary hemorrhages, sequestered parasitized red blood cells (PRBC), neutrophils, and monocytes containing malarial pigment in lung tissue (20). Nevertheless, the biological process that triggers MA-RD is not clear. In this way, animal models have been an indispensable tool to understand lung dysfunction during malaria. However, since most experimental studies did not evaluate all factors that characterize ARDS, it is more appropriate to use the term malaria-induced acute lung injury (MA-ALI) to depict experimental results. Unlike cerebral malaria, which is mainly studied in *P. berghei*-infected C57BL/6 mice (21), lung malaria can be observed in *P. berghei* ANKA-infected C57BL/6 (22, 23), *P. berghei* NK65-infected C57BL/6 (24, 25), *P. berghei* ANKA-infected DBA mice (26), *P. berghei* ANKA-infected CBA mice (27), among others (28). These models show that malaria-induced experimental lung dysfunction is characterized by vascular dysfunction induced by CD8^+^ T cells, presence of PRBC, hemorrhages, neutrophils, and monocytes containing malarial pigment. On the other hand, it has been shown that, at 24 h after infection, a time point at which inflammatory mediators are not yet detected, it is possible to observe PRBC, neutrophils, and mononuclear cells in the lung tissue (29, 30). Thus, it is unclear whether inflammatory cells, PRBC, blood, and pigment from malaria are a consequence or trigger the pulmonary pathology seen during malaria.

## The Role of Heme Derivatives in Lung Pathology During Malaria

The study of heme and its derivatives in MA-RD is not elementary. The complex named heme (protoporphyrin IX + Fe II) is an important cofactor in several biological processes such as oxygen transfer, storage and activation, and electron transfer (31). During the respiratory process, the hemoglobin (Hb) containing heme captures and releases the oxygen without modifying iron oxidative state (32). However, 1–3% of Hb undergoes auto-oxidation, and oxygen is reduced to superoxide anion (${\text{O}}_{2}^{•-}$) and generates methemoglobin [Hb plus hemin (Fe III)] (32).

**Figure 1:**
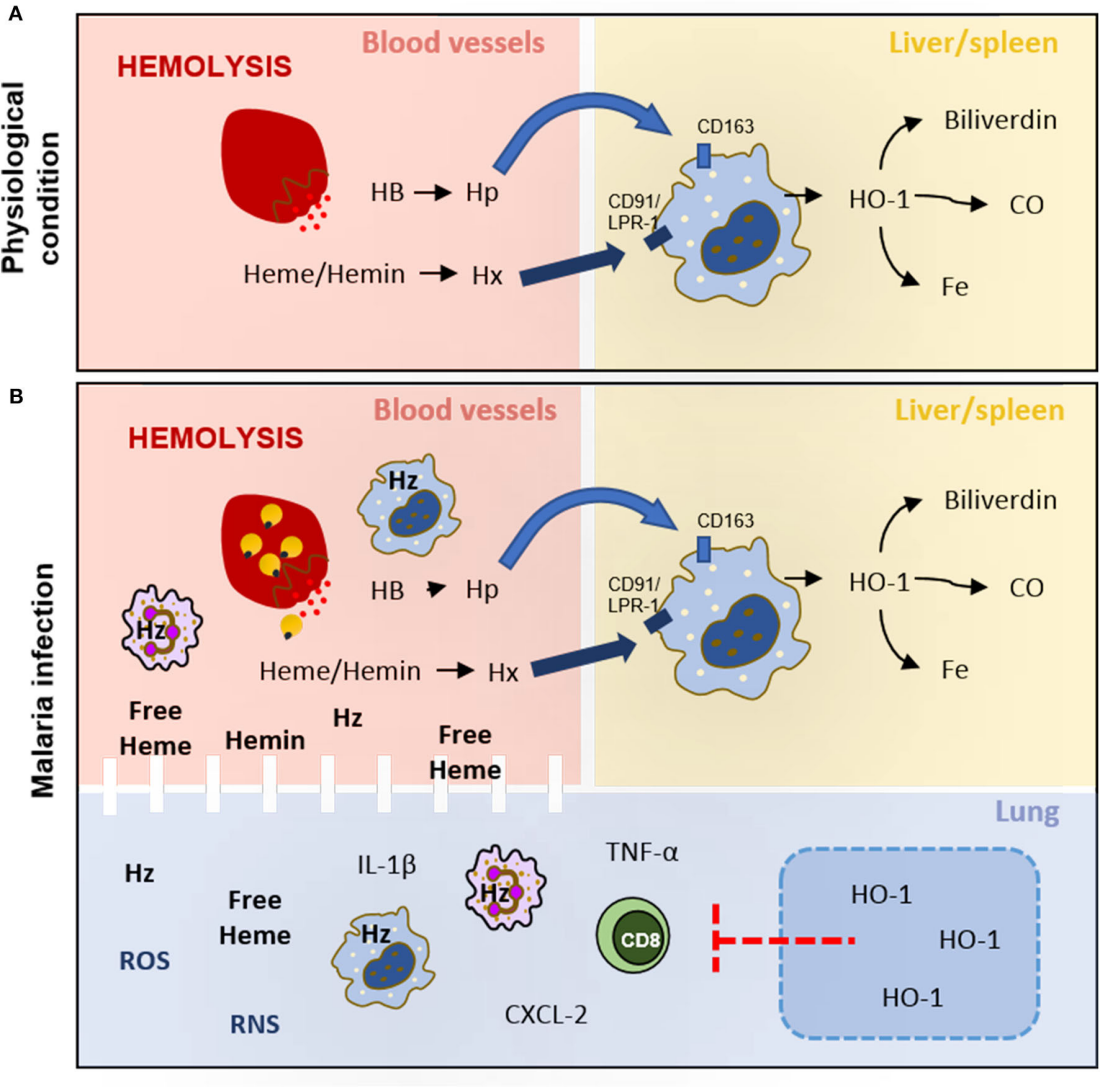
Hemolysis under physiological conditions and malaria infection. **(A)** During hemolysis, hemoglobin (HB) and free heme/hemin are captured by haptoglobin (Hp) and hemopexin (Hx), respectively, in blood vessels. These complexes (HB-Hp and Heme-Hx) target macrophages CD163^+^ and CD91/LRP-1^+^ in the liver and spleen to be metabolized by heme oxygenase 1 (HO-1) to biliverdin, carbon monoxide (CO), and iron (Fe). **(B)** The hemolysis increases during the release of merozoites saturates the activity of haptoglobin (Hp) and hemopexin (Hx), leading to heme, hemin, and hemozoin (Hz) circulating in plasma. These heme derivatives increase ROS and RNS production and activate leukocytes to produce cytokines and chemokines that damage lung tissue and endothelial barriers. HO-1 induction would decrease leukocyte activation and migration, reduce inflammatory mediators production, and restore the integrity of the endothelial cell barrier in lung tissue.

Heme and its analogs localize differently on erythrocyte membranes and exhibit distinct roles in its partitioning, leakage, and fusion (33). Under physiological conditions, when intravascular hemolysis occurs during the destruction of senescent erythrocytes and/or enucleation of erythroblasts, some hemoglobin, free heme, or hemin can be released into the plasma where they bind to soluble haptoglobin (Hp) or hemopexin (Hx) (reviewed by 27; 28). In the liver, the complexes are recognized by specific receptors on Kupffer cells such as CD163 and CD91/LRP-1, respectively, and metabolized by heme oxygenase-1 (HO-1) to iron, carbon monoxide, and biliverdin that will be stored or act as antioxidant molecules (34–36) (Figure 1A).

However, in hemolytic diseases, intravascular hemolysis increases, becoming a serious pathological complication (37). During the intraerythrocytic stage, parasites lyse the erythrocyte to release merozoites that rapidly invade new host cells (38). This lytic process also releases the infected red blood cell contents into the host bloodstream, including undigested hemoglobin, free heme, and hemozoin. The augment of extracellular levels of hemoglobin may reduce levels of available free Hp, making this pathway ineffective (39), while the large content of heme and hemin circulating in plasma can exhaust the binding capacity of Hx and their metabolism by HO-1. These events result in the increase in oxidation from heme to hemin and consequently methemoglobin (hemoglobin plus hemin) formation (40). It is important to note that the binding affinity of globin to hemin is weak and can lead to free heme release (32). The free heme leads to oxidative damage by the generation of reactive oxygen species (ROS) [e.g., superoxide (${\text{O}}_{2}^{•-}$), hydrogen peroxide (H_2_O_2_), and hydroxyl radical (HO∙)], reactive nitrogen species (RNS) (e.g., nitric oxide (∙NO), nitrogen dioxide (∙NO_2_)], and peroxynitrite (ONOO^−^) (41). These reactive species mediate the activation of inflammatory pathways and tissue damage, in addition to the loss of erythrocyte deformation ability and the endothelial barrier integrity by inducing lipid peroxidation of the membrane (42–44). Therefore, the consequences of heme derivative release might depend on their concentration and the environment in which they are found (38, 40).

As mentioned above, malaria is a hemolytic disease; thus, free heme and hemin released during hemolysis due to erythrocyte rupture during the plasmodium life cycle exert effects that contribute to malaria pathology. Beyond the free heme and hemin, heme can also be found in a biocrystallized structure named hemozoin in the *Plasmodium* sp. *Plasmodium* digest ~65% of total erythrocyte hemoglobin during intraerythrocytic development. Part of the hemoglobin's amino acids is incorporated in parasite proteins; however, since free heme released during hemoglobin digestion is a toxic by-product, *Plasmodium* biocrystallizes heme to hemozoin to store it as a nontoxic molecule in the digestive vacuole (45, 46).

The erythrocyte content (cytoplasm, parasite components, hemozoin, and free heme) released during hemolysis is engulfed by phagocytes such as macrophages, neutrophils, and dendritic cells (47). The accumulation of hemozoin in these immune phagocytic cells reflects the parasite burden and coincides with periodic fevers and high circulating levels of proinflammatory cytokines. In this way, this pigment is used to measure malaria severity and identify parasite developmental stages (6). Adult patients who died of severe *P. falciparum* malaria had significantly higher proportions of neutrophils and monocytes containing hemozoin than surviving patients (48). In addition, patients with MA-RD demonstrated high lung deposition of hemozoin and internal alveolar hemorrhage compared with those with non-MA-RD lungs (49). The same group also showed that hemozoin leads to loss of alveolar integrity by increasing the production of interleukin (IL)-1β by monocytes, which induces pneumocytes type II apoptosis (50). These observations are also seen in mouse lungs. The C57BL/6 mice infected with *P. berghei* NK65 showed a grayish-brown discoloration due to hemorrhages and hemozoin deposition, in addition to tissue edema and a marked inflammatory cells influx (24, 51).

The use of purified hemozoin has already been proposed to access its pathological role *in vitro* and *in vivo* (52). However, the method used to extract hemozoin is not effective in purifying it, since biological effects observed were attributed to a DNA contamination in hemozoin extract (53). In this context, some authors resort to the use of β-hematin, a compound produced *in vitro* using parasite lysate to provide necessary enzymes to biocrystallization. However, the artificial process to produce β-hematin results in substances different in shape and size from the natural ones, which could mask the results [reviewed in (46)]. Despite the immunological activity of synthetic hemozoin being controversial, several studies have demonstrated that both parasite-derived hemozoin and synthetically produced hemozoin, once phagocytized, activates both mouse and human leukocytes to produce proinflammatory cytokines such as tumor necrosis factor alpha (TNF-α) and IL-1β (54) and macrophage inflammatory protein (MIP)-1α/CCL3, MIP-1β/CCL4, MIP-2/C-X-C Motif Chemokine Ligand 2 (CXCL2), and MCP-1/CCL2 chemokines through oxidative stress-dependent and stress-independent mechanisms (55). Besides, Huy and coworkers showed that the treatment with β-hematin increased myeloperoxidase activity of peritoneal cells *in vivo* and neutrophil chemotaxis *in vitro* (56).

Despite the compelling data showing the deleterious effects of heme during malaria, in the last decade, several studies have shown that heme pathway could be beneficial to host outcomes. Balb/c mice, a resistant strain to multiorgan dysfunction (MOD) triggered by *P. berghei-*ANKA infection, expressed HO-1 in brain tissue during *P. berghei* infection. In addition, HO-1 knockout Balb/c mice succumb to *P. berghei* infection, through a mechanism that can be reversed by CD8^+^ T cell depletion, which suggests that heme metabolism is involved in malaria resistance by modulating immunological response. Interestingly, studies with C57BL/6 mice, a susceptible strain to MOD triggered by *P. berghei*-ANKA infection, also produced HO-1 in brain tissue, however, correlated with parasite inoculum. It is well established that parasite inoculum modulates disease outcome (57). Additionally, the 10^5^ *P. berghei* pRBC inoculum did not induce HO-1 expression in the brain tissue (58), while the 10^6^ *P. berghei* pRBC inoculum induces HO-1 expression in the brain 4 days postinfection (59). It is noteworthy that parasite inoculum did not interfere in increased levels of free heme in plasma, which suggests that heme in malaria-susceptible hosts is not enough to induce HO-1; HO-1 is insufficiently produced/activated to induce free heme clearance, or the produced HO-1 is saturated. For instance, C57BL/6 *P. berghei*-infected mice treated with cobalt protoporphyrin, a pharmacological intervention that stimulates HO-1 production and activity, reduced brain edema and microvascular congestion (58). The authors also gave CO, a downstream metabolite in the heme clearance pathway, and further observed a reduction in CD8^+^ T cells in the brain tissue, showing that the appropriate amounts of HO-1 are effective to protect susceptible mice from MOD. In accordance, the balance between free heme and HO-1 production is important to improve the outcome of *P. berghei*-infected mice that carry hemoglobin beta-chain mutation, named sickle Hb (HbS). The authors observed that mice with HbS phenotype did not develop cerebral malaria by two different mechanisms, and both pathways depend on low levels of free heme on the bloodstream. The first mechanism involves the stimulation of HO-1 production, and the second one involves heme-induced immunoregulatory roles. The authors suggest that there is a pathogenic and a protective concentration of circulating free heme during malaria (60).

In recent reviews by Frimat et al. (61) and Immenschuh et al. (40), the heme clearance pathway has been proposed as targets to treat hemolytic diseases. Frimat suggests that two different approaches should be considered to treat hemolytic disease: first, by modulating molecules from the heme clearance pathway as by administering hemopexin or inducing HO-1, and second, by treating oxidative stress and inflammation induced by heme (61). In addition, Immenschuh and colleagues state that heme exerts different effects depending on the target cell. Endothelial cells rapidly respond to heme by means of HO-1 production, which suggests that the lung, as a highly vascularized organ, is an important organ to study therapeutic interventions aiming at heme clearance. Compounds such as desoxyrhapontigenin, statins, curcumin, hemin, quercetin, and cobalt protoporphyrin have already been used to attenuate experimental lung dysfunction by inducing HO-1 expression (40). However, few studies have been dedicated to assessing whether HO-1 induction would attenuate malaria-induced ALI. Pereira et al. (62), using the *P. berghei*-infected DBA/2 mice model of MA-ALI, gave hemin to infected mice and observed an increase in HO-1 production correlated with attenuation of lung dysfunction and inflammatory response associated to alteration in lung histoarchitecture. As well, Liu et al. (59) showed that HO-1 expression in the lung tissue during experimental malaria depends on CXCL10 and signal transducer and activator of transcription 3 (STAT3). The authors further show that free heme is detectable in plasma since the second day of infection. At the same time point, they also observed HO-1 expression in the lung tissue but not in the brain tissue, supporting the idea that the lung is one of the most important organs for the heme clearance pathway (Figure 1B).

Thus, considering the biological mechanism by which HO-1 induction attenuates brain dysfunction during experimental cerebral malaria, we can speculate that during experimental MA-ALI, the induction of HO-1 downmodulates CD8^+^ T cell activation and migration to lung tissue, reduces the production of inflammatory mediators, and restores endothelial cell barrier integrity.

## Conclusion

Free heme and heme derivatives have been widely recognized as pathological molecules in several hemolytic conditions. The participation of heme in malaria is very peculiar because it exerts its effects through different molecular structures as free heme, hemin, and hemozoin. Several studies concerning malaria-induced lung dysfunction show that heme derivatives affect alveolar integrity, induce the production of inflammatory mediators, and accumulate the inflammatory cells in the lung tissue. On the other hand, more recent studies propose that heme exerts a beneficial role during malaria infection by inducing cytoprotective pathways such as HO-1 production. Indeed, more studies are necessary to define the role of heme during malaria-induced lung dysfunction. Overall, we can conclude that the imbalance between free concentration, production/saturation of HO-1, and the activation of coexisting anti-inflammatory pathways dictate if heme is a friend or foe to malaria patients.

## Author Contributions

TP and MS wrote the manuscript. All authors contributed to the article and approved the submitted version.

## Conflict of Interest

The authors declare that the research was conducted in the absence of any commercial or financial relationships that could be construed as a potential conflict of interest.
